# Pleiotropic Properties of Valsartan: Do They Result from the Antiglycooxidant Activity? Literature Review and *In Vitro* Study

**DOI:** 10.1155/2021/5575545

**Published:** 2021-03-03

**Authors:** Kacper Maksymilian Mil, Małgorzata Ewa Gryciuk, Cezary Pawlukianiec, Małgorzata Żendzian-Piotrowska, Jerzy Robert Ładny, Anna Zalewska, Mateusz Maciejczyk

**Affiliations:** ^1^Students Scientific Club “Biochemistry of Civilization Diseases” at the Department of Hygiene, Epidemiology and Ergonomics, Medical University of Bialystok, 2c Mickiewicza Street, 15-233 Białystok, Poland; ^2^Department of Hygiene, Epidemiology and Ergonomics, Medical University of Bialystok, 2c Mickiewicza Street, 15-233 Białystok, Poland; ^3^1st Department of General Surgery and Endocrinology, Medical University of Bialystok, 24a M. Sklodowskiej-Curie Street, 15-274 Białystok, Poland; ^4^Experimental Dentistry Laboratory, Medical University of Bialystok, 24a M. Sklodowskiej-Curie Street, 15-274 Białystok, Poland

## Abstract

Valsartan belongs to angiotensin II type 1 (AT1) receptor blockers (ARB) used in cardiovascular diseases like heart failure and hypertension. Except for its AT1-antagonism, another mechanism of drug action has been suggested in recent research. One of the supposed actions refers to the positive impact on redox balance and reducing protein glycation. Our study is aimed at assessing the antiglycooxidant properties of valsartan in an *in vitro* model of oxidized bovine serum albumin (BSA). Glucose, fructose, ribose, glyoxal (GO), methylglyoxal (MGO), and chloramine T were used as glycation or oxidation agents. Protein oxidation products (total thiols, protein carbonyls (PC), and advanced oxidation protein products (AOPP)), glycooxidation products (tryptophan, kynurenine, N-formylkynurenine, and dityrosine), glycation products (amyloid-*β* structure, fructosamine, and advanced glycation end products (AGE)), and albumin antioxidant activity (total antioxidant capacity (TAC), DPPH assay, and ferric reducing antioxidant power (FRAP)) were measured in each sample. In the presence of valsartan, concentrations of protein oxidation and glycation products were significantly lower comparing to control. Moreover, albumin antioxidant activity was significantly higher in those samples. The drug's action was comparable to renowned antiglycation agents and antioxidants, e.g., aminoguanidine, metformin, Trolox, N-acetylcysteine, or alpha-lipoic acid. The conducted experiment proves that valsartan can ameliorate protein glycation and oxidation *in vitro* in various conditions. Available animal and clinical studies uphold this statement, but further research is needed to confirm it, as reduction of protein oxidation and glycation may prevent cardiovascular disease development.

## 1. Introduction

The formation of free radicals is an inevitable consequence of aerobic metabolism. As long as physiological mechanisms manage to eliminate reactive oxygen species (ROS), their adverse action on structural proteins, enzymes, membrane lipids, or nucleic acids may remain unseen [1]. However, the generation of ROS may exceed the capability of the organism to neutralize them. This situation is called redox imbalance and leads to oxidative damage to cellular biomolecules [2]. Indeed, ROS are not only proven to play a crucial role in many physiological processes [3, 4], but they are also a vital factor in the pathogenesis of many diseases like obesity [5, 6], insulin resistance [7, 8], hypertension [9, 10], and chronic heart failure [11, 12].

ROS's role in the pathogenesis of hypertension is complicated and involves many different biochemical mechanisms [13–15]. ROS produced by nicotinamide adenine dinucleotide phosphate (NADPH) oxidases are responsible for nitric oxide (NO) depletion caused by its interaction with superoxide radical anions (O_2_^-•^). This results in decreased endothelial nitric oxide synthase (eNOS) activity and leads to endothelial dysfunction, which is considered one of the essential pathogenetic factors [16, 17]. What is more, oxygen-free radicals can act on redox-sensitive genes in vascular smooth muscle cells and promote their mitogenic phenotype, stimulate fibrosis by inhibiting matrix metalloproteinases, or ameliorate an arterial wall calcification by activation of specific bone morphogenetic proteins (BMP). As an effect of this remodeling, stiffness of the vessel and peripheral resistance increase [9].

It is also reported that glycation of vascular proteins plays a vital role in hypertension development [5, 13, 14]. Interestingly, higher circulating levels of advanced glycation end products (AGE) correlate with increased arterial stiffness [18, 19]. Indeed, AGE accumulation in blood vessels impairs endothelial function by decreasing eNOS activity and reducing NO bioavailability [14, 20]. AGE also combine with a specific receptor (RAGE) to activate the transcription factor NF-*κ*B (nuclear factor kappa-light-chain-enhancer of activated B cell). This not only stimulates ROS production but also activates many proinflammatory genes such as interleukins (IL-1b, IL-2, IL-6, and IL-8), adhesion molecules (vascular cell adhesion protein 1 (VCAM-1), intercellular cell adhesion protein 1 (ICAM-1)), growth and differentiation factors (vascular endothelial growth factor (VEGF), and transforming growth factor *β*2 (TGF-*β*2)) [20, 21]. Under these conditions, ROS production is intensified, which increases the already existing oxidative stress. Nevertheless, AGE also affect the activation of monocytes that overexpress the CD36 sweeping receptor. In this way, lipoproteins foam cells are formed, and atherosclerosis development is accelerated [20, 21]. Therefore, the use of antioxidants in cardiovascular diseases may have several positive effects [22].

According to recent studies, the drug which may potentially exhibit antioxidant and antiglycation activity is valsartan. Valsartan, (2S)-3-methyl-2-[pentanoyl-[[4-[2-(2H-tetrazole-5-yl)phenyl]phenyl]methyl]amino]butanoic acid, is a lipophilic, nonpeptide, tetrazole derivative, a selective antagonist of angiotensin II type 1 receptor (AT_1_), which is widely used in many cardiovascular conditions like hypertension or chronic heart failure. The drug is administered orally, is characterized by rapid absorption and low bioavailability (25%), and is almost entirely bound to plasma proteins (94-97%) [23, 24]. The hepatic products of the metabolism of valsartan are mainly excreted with bile [25]. In comparison to other antihypertensives, valsartan is well tolerated and deprived of many side effects, like cough or angioedema, characteristic for angiotensin-converting enzyme inhibitors (ACEI). An occurrence of valsartan side effects in clinical studies was comparable to placebo, which confirms the drug's safety [26]. Moreover, it has been proven to act nephroprotective in patients with diabetes mellitus and chronic renal failure and significantly reduce albuminuria [27, 28]. Some reports are claiming that valsartan may present additional mechanisms of action besides its AT_1_ receptor antagonism. It was proven that patients treated with valsartan presented less urinary oxidative stress markers [29]. Similar outcomes were observed in mice where valsartan diminishes oxidative damage and acts nephroprotective [30].

However, it is still uncertain whether valsartan has antioxidant and antiglycation activity. Indeed, the impact of various glycating agents on protein oxidation/glycation in *in vitro* or *in vivo* models has not yet been evaluated. The effect of valsartan action was also not compared to other substances with recognized antioxidant properties. This is particularly important because confirmation of the antiglycooxidant activity of valsartan may result in a revision of the guidelines for the use of the drug in cardiovascular disease and diabetes, making it the first-line medication in patients with enhanced protein glycation. For this reason, we conducted a study to assess valsartan's effect on protein oxidation, glycation, and total antioxidant activity in *in vitro* model of oxidized bovine serum albumin (BSA). Either oxidizing (chloramine T) or glycating agents (glucose, fructose, ribose, glyoxal, and methylglyoxal), as well as antioxidants (Trolox, N-acetylcysteine, lipoic acid, and captopril) and protein glycation inhibitors (aminoguanidine and metformin), were used to compare the results of antiglycooxidant capabilities of valsartan [31–35]. We assessed the concentrations of protein oxidation products (total thiols, protein carbonyls, and advanced oxidation protein products (AOPP)), glycooxidation products (tryptophan, kynurenine, N-formylkynurenine, and dityrosine), levels of albumin glycation products (amyloid-*β* structure, fructosamine, and advanced glycation end products (AGE)), and antioxidant potential of albumin (total antioxidant capacity (TAC), DPPH assay, and ferric reducing antioxidant power (FRAP)). We have also made a detailed literature review on the antiglycooxidant properties of valsartan.

## 2. Methods

### 2.1. Reagents and Equipment

All reagents (analytical grade) were purchased from Sigma-Aldrich (Nümbrecht, Germany, or Saint Louis, MO, USA). Solutions were sterilized by filtration through 0.2 mm membrane filters directly before use. The fluorescence and absorbance were evaluated using a microplate reader (M200 PRO multimode microplate reader; Tecan Group Ltd., Männedorf, Switzerland).

### 2.2. Experimental Model

The glycated/oxidated BSA formation was implemented according to the previously published method [31–36]. BSA, of 96% purity, was dissolved in sodium phosphate buffer (1 M, pH 7.4), which contained 0.02% sodium azide as a preservative.

Chloramine T was used as an oxidation agent. 0.09 mM BSA and 1 mM valsartan were incubated with 20 mM chloramine T for 60 minutes [37, 38].

As glycating agents, sugars (glucose (Glu), fructose (Fru), and ribose (Rib)) and aldehydes (glyoxal (GO), and methylglyoxal (MGO)) were used. To assess the additives' effect on protein glycation, 0.09 mM BSA and 1 mM valsartan were incubated with 0.5 M Glu, Fru, and Rib for 6 days or 2.5 mM GO and MGO for 12 hours [31, 32, 39]. GO and MGO were used within a month after delivery, and solutions were prepared immediately before use [34].

Incubation was conducted in the closed vials, darkly, with continuous shaking (50 rpm) [31–35]. These conditions and concentrations of glycating agents were validated based on previously published kinetic studies [31, 34].

Captopril, Trolox, N-acetylcysteine (NAC), and lipoic acid (ALA) were used as protein oxidation inhibitors, while aminoguanidine and metformin as inhibitors of protein glycation [31–36, 38]. The concentration of all additives was 1 mM, which was based on the kinetic studies, in proportion to the high concentrations of the glycating agents [31, 32, 34, 35, 37, 39–41].

All experiments were performed three times, each time in duplicate.

### 2.3. Protein Oxidation Products

Total thiols were detected using Ellman's reagent by the colorimetric method [42]. The absorbance was measured at 412 nm wavelength. Thiol groups' content was established based on a standard curve for N-acetylcysteine (NAC) [42].

The carbonyl groups' reaction with 2,4-dinitrophenylhydrazine (2,4-DNPH) was performed to examine protein carbonyl concentration in proteins that underwent oxidative damage. The absorbance of the products of this reaction was evaluated colorimetrically at 355 nm wavelength [43].

The concentration of advanced oxidation protein products was measured by spectrophotometric detection. 200 *μ*L of examined solutions diluted with PBS in 1 : 5 ratio (*v* : *v*), chloramine T standard solutions (0-100 *μ*mol/L), and 200 *μ*L of blank PBS were placed in 96-well microplates. Then, 20 *μ*L of acetic acid and 10 *μ*L of 1.16 M potassium iodide were added to wells. Immediately after that, the absorbance was measured at 340 nm wavelength concerning blank (200 *μ*L PBS, 20 *μ*L acetic acid, and 10 *μ*L potassium iodide). Chloramine T solutions presented linear absorbance in the range of 0-100 *μ*mol/L [44].

### 2.4. Protein Glycooxidation Products

Tryptophan, kynurenine, N-formylkynurenine, and dityrosine were evaluated by measuring emission and excitation at 95/340, 365/480, 325/434, and 330/415 nm, respectively. The samples were diluted with 0.1 M sulfuric acid in a 1 : 5 ratio (*v* : *v*). Results were standardized to the fluorescence of 0.1 mg/mL quinine sulfate in 0.1 M sulfuric acid [45].

### 2.5. Protein Glycation Products

The assay was performed to measure fluorescence emitted when amyloid fibrils or oligomers are bound to amyloid-*β* structure. 10 *μ*L of Thioflavin T and 90 *μ*L of samples were mixed and transferred to a microplate; then, the fluorescence was assessed at 385/485 nm [46, 47].

The fructosamine content was detected colorimetrically with nitro blue tetrazolium (NBT) assay. The absorbance was measured at 525 nm using the monoformazan extinction coefficient (12.640 M^−1^ m^−1^) [48].

All examined samples were diluted with 0.1 M sulfuric acid (1 : 5, *v* : *v*) [18]. Then, the content of advanced glycation end products (AGE) was measured using the spectrophotometric method at 440/370 nm at a 96-well microplate reader [44, 49]. AGE content was also analyzed with the ELISA method (UCSN, Life Science, Wuhan, China).

### 2.6. Antioxidant Activity

TAC of each sample was measured according to Erel's method. For this purpose, 2,2-azinobis(3-ethylbenzene-thiazoline-6-sulfonate) (ABTS) radical cation decolorization assay was used. ABTS^+^ was obtained by reacting ABTS with potassium persulfate and incubated for 12 hours at room temperature. After mixing 10 *μ*L of samples with 1 mL of ABTS^+^, the absorbance readings were taken at 735 nm. Results of decolorization were linear with increasing Trolox concentrations [50, 51].

The determination of free radicals scavenging activity was performed according to the Brand–Williams method. 10 *μ*L of each sample and 390 *μ*L methanolic diluted DPPH were mixed and placed on a 96-well microplate. The solutions were incubated in darkness at room temperature for 30 minutes. The absorbance was measured at 515 nm [52, 53].

The ferric reducing antioxidant power of each sample was measured following the Benzie and Strain method. FRAP reagent was prepared by mixing 25 mL acetate buffer, 2.5 mL FeCl_3_·6H_2_O solution, and 2.5 mL TPTZ solution and warming the solution to 37°C. 10 *μ*L of sample and 300 *μ*L of FRAP reagent were diluted with 30 *μ*L of water and transferred to 96-wells microplate. The change of absorbance was calculated for each sample and related to the absorbance of Fe^II^ standard solution [54].

### 2.7. Statistical Analysis

The statistical analysis was conducted using GraphPad Prism 8.3.0 (GraphPad Software, La Jolla, CA, USA). The results were expressed as a percentage of the corresponding control values (BSA + glycating/oxidizing agent). Differences between groups were assessed by one-way ANOVA followed by Tukey's post hoc test for multiple comparisons. *p* < 0.05 was considered statistically significant. Multiplicity adjusted *p* value was also calculated.

## 3. Results

### 3.1. The Impact of Valsartan and Other Additives on Protein Oxidation Products, Glycooxidation Products, and Glycation Products as well as Antioxidant Activity in Glucose- (Glu-) Induced Albumin Glycation

The addition of glucose to the BSA solution led to increased protein oxidation products—PC and AOPP. Decreased levels of aforementioned parameters were noticed when BSA + Glu (control) were incubated with valsartan (84%, 79% compared to control, respectively), NAC (76%, 76% vs. control), ALA (69%, 76% vs. control), captopril (78%, 77% vs. control), or aminoguanidine (77%, 68% vs. control). Moreover, in samples containing Trolox (109% vs. control), NAC (119% vs. control), ALA (105% vs. control), or aminoguanidine (116% vs. control), significantly higher values of total thiols were observed (Table 1).

The presence of glucose in the BSA sample caused a significant increase in kynurenine levels, N-formylkynurenine, and dityrosine due to albumin glycooxidation. In contrast, tryptophan concentration was lower in BSA + Glu compared to BSA alone. The addition of valsartan (70%, 71%, and 55% vs. control, respectively) and other additives resulted in decreased kynurenine contents, N-formylkynurenine, and dityrosine comparing to control. Moreover, statistically higher tryptophan (valsartan: 114% vs. control) in all investigated samples versus control was observed (Table 1).

Amyloid-*β* structure, fructosamine, and AGE contents were significantly higher in BSA incubated with glucose than BSA alone. All reviewed substances caused a significant decrease in parameters above (valsartan: 89%, 56%, and 73% vs. control, respectively), excluding sample with metformin in AGE measurement (Table 1).

Glucose was also responsible for a decreased antioxidant activity (TAC, DPPH, and FRAP). The analysis of these parameters showed significantly greater DPPH and FRAP in BSA + Glu + valsartan (109%, 105% vs. control, respectively), Trolox (109%, 113% vs. control), NAC (128%, 122% vs. control), ALA (115%, 111% vs. control), captopril (111%, 114% vs. control), or aminoguanidine (111%, 113% vs. control) comparing to control. Furthermore, the addition of valsartan (107% vs. control), NAC (114% vs. control), and aminoguanidine (110% vs. control) resulted in increased TAC compared to control (Table 1).

### 3.2. The Impact of Valsartan and Other Additives on Protein Oxidation Products, Glycooxidation Products and Glycation Products as well as Antioxidant Activity in Fructose- (Fru-) Induced Albumin Glycation

Fructose-induced protein oxidation resulted in increased PC and AOPP levels and decreased total thiols level compared to BSA alone. All investigated agents caused a significant decrease in concentrations of PC and AOPP (valsartan: 81%, 58% vs. control, respectively), excluding BSA + Fru + metformin in AOPP concentration. Moreover, the addition of valsartan (105% vs. control), NAC (114% vs. control), ALA (107% vs. control), and aminoguanidine (105% vs. control) led to a higher concentration of total thiols compared to control (Table 2).

The results of protein glycooxidation products measurement showed significantly higher concentrations of kynurenine, N-formylkynurenine, and dityrosine in BSA + Fru comparing to BSA alone. However, the content of tryptophan decreased after fructose was added. In samples with all reviewed inhibitors, significantly lower kynurenine concentrations, N-formylkynurenine, and dityrosine (valsartan: 67%, 57%, and 65% vs. control, respectively) were observed (Table 2).

Moreover, the presence of fructose in the BSA solution resulted in greater concentrations of measured glycation products. The analysis showed significantly lower levels of amyloid-*β* structure, fructosamine, and AGE in samples with the addition of all investigated protein glycation and oxidation inhibitors (valsartan: 88%, 53%, and 84% vs. control, respectively) comparing to control.

When BSA was incubated with fructose, significantly decreased TAC, DPPH, and FRAP were noticed. The addition of the antiglycooxidative agents such as NAC (146%, 129% vs. control, respectively), ALA (129%, 108% vs. control), and captopril (133%, 108% vs. control) resulted in higher levels of DPPH and FRAP. Antioxidant properties were shown by valsartan as well (DPPH: 126%, FRAP: 104% vs. control). Interestingly, a significantly decreased FRAP level in BSA + Fru + Trolox (95% vs. control) compared to BSA + Fru was observed (Table 2).

### 3.3. The Impact of Valsartan and Other Additives on Protein Oxidation Products, Glycooxidation Products, and Glycation Products as well as Antioxidant Activity in Ribose- (Rib-) Induced Albumin Glycation

The addition of ribose to BSA solution resulted in lower total thiols level and higher PC and AOPP levels. All reviewed agents (valsartan: 68%, 76% vs. control, respectively) caused a significant decrease in PC and AOPP contents. Moreover, after inhibitors (valsartan: 105% vs. control) were added to BSA + Rib, statistically higher total thiols were noticed (Table 3).

Assays evaluating glycooxidation products revealed that ribose caused an increase of kynurenine, N-formylkynurenine, and dityrosine levels. Significantly greater concentrations of tryptophan were observed in comparison to BSA + Rib when BSA + Rib were incubated with valsartan (114% vs. control), Trolox (103% vs. control), NAC (105% vs. control), ALA (113% vs. control), captopril (106% vs. control), and aminoguanidine (118% vs. control). There were also significantly lower kynurenine values, N-formylkynurenine, and dityrosine in samples with agents above (valsartan: 83%, 76%, and 68% vs. control, respectively) (Table 3).

Regarding glycation products, the presence of ribose in BSA resulted in greater amyloid-*β* structure, fructosamine, and AGE compared to BSA alone. Furthermore, decreased levels of these parameters were noticed in samples with BSA + Rib and all investigated inhibitors (valsartan: 96%, 58%, and 66% vs. control, respectively), excepting metformin in amyloid-*β* structure level (Table 3).

Moreover, decreased antioxidant activity was present when ribose was incubated with BSA compared to BSA alone. The addition of antiglycooxidative agents (valsartan: 111%, 119%, and 106% vs. control), excluding metformin, resulted in significantly increased TAC, DPPH, and FRAP (Table 3).

### 3.4. The Impact of Valsartan and Other Additives on Protein Oxidation Products, Glycooxidation Products, and Glycation Products as well as Antioxidant Activity in Glyoxal- (GO-) Induced Albumin Glycation

Glyoxal-induced protein oxidation resulted in increased PC and AOPP and decreased total thiols, similarly to carbohydrates described previously. Samples with all investigated agents were characterized by lower levels of PC and AOPP (valsartan: 68%, 40% vs. control, respectively). Significantly higher total thiols were observed in presence of valsartan (103% vs. control), Trolox (108% vs. control), NAC (118% vs. control), captopril (114% vs. control), aminoguanidine (114% vs. control), and metformin (102% vs. control) (Table 4).

The glycooxidation products analysis revealed that glyoxal addition increased kynurenine, N-formylkynurenine, and dityrosine concentrations in BSA + GO samples. Furthermore, all reviewed inhibitors caused a significant increase in the aforementioned parameters (valsartan: 83%, 42%, and 72% vs. control, respectively). However, concentrations of tryptophan were higher in the presence of all substances compared to control (valsartan: 133% vs. control) (Table 4).

The presence of glyoxal in BSA solution resulted in higher glycation product content. Moreover, decreased amyloid-*β* structure, fructosamine, and AGE concentrations in all investigated samples versus control were observed (valsartan: 71%, 87%, and 52% vs. control, respectively), excepting sample with metformin (Table 4).

Regarding total antioxidant potential assays, the BSA + GO sample was characterized by significantly lower TAC, DPPH, and FRAP parameters than the BSA alone sample. Significantly greater TAC and DPPH values in all investigated samples, excluding DPPH in a sample with captopril, were observed. The addition of valsartan (118% vs. control), NAC (117% vs. control), ALA (107% vs. control), and aminoguanidine (124% vs. control) caused an increase of FRAP compared to control. On the other hand, the presence of captopril (88% vs. control) and metformin (78% vs. control) led to significantly lower FRAP value (Table 4).

### 3.5. The Impact of Valsartan and Other Additives on Protein Oxidation Products, Glycooxidation Products, and Glycation Products as well as Antioxidant Activity in Methylglyoxal- (MGO-) Induced Albumin Glycation

Methylglyoxal induced oxidative damage and significantly greater PC and AOPP levels in BSA samples. All investigated substances (valsartan: 66%, 55% vs. control, respectively) diminished this action and decreased the parameters above (except metformin). Moreover, significantly higher total thiols concentrations were noticed when valsartan (106% vs. control), Trolox (107% vs. control), NAC (109% vs. control), captopril (107% vs. control), and aminoguanidine (105% vs. control) were added (Table 5).

The presence of methylglyoxal resulted in a significant decrease in tryptophan concentration and an increase of kynurenine, N-formylkynurenine, and dityrosine concentrations. Significantly higher tryptophan levels were observed after inhibitors were added (valsartan: 107% vs. control). The majority of investigated substances (valsartan: 87%, 74%, and 72% vs. control, respectively) caused a significant decrease of kynurenine, N-formylkynurenine, and dityrosine concentrations (except ALA and metformin) (Table 5).

Furthermore, a decrease of amyloid-*β* structure, fructosamine, and AGE concentrations was observed due to methylglyoxal addition to the BSA sample. All used inhibitors (valsartan: 66%, 38%, and 56% vs. control, respectively) were responsible for the decrease of given parameters (Table 5).

Methylglyoxal was also the cause of decreased TAC, DPPH, and FRAP values compared to BSA alone. All the investigated samples (valsartan: 144%, 113%, and 132% vs. control, respectively), except metformin, were characterized by significantly higher TAC levels in comparison to control (Table 5).

### 3.6. The Impact of Valsartan and Other Additives on Protein Oxidation Products, Glycooxidation Products, and Glycation Products as well as Antioxidant Activity in Chloramine T-Induced Albumin Glycation

Markers of oxidative damage (PC and AOPP) were significantly greater in the presence of chloramine T compared to BSA alone. In all investigated samples (valsartan: 71%, 56% vs. control, respectively), significantly decreased levels of the parameters above versus control were observed. In addition, all used inhibitors (valsartan: 110% vs. control) caused a significant increase of total thiols level (Table 6).

Glycooxidation of proteins induced by chloramine T resulted in greater kynurenine concentrations, N-formylkynurenine, and dityrosine together with a lower concentration of tryptophan. The addition of valsartan (89%, 69% vs. control, respectively) and other substances led to decreased amounts of given parameters compared to control. In contrast, all the additives (excluding aminoguanidine) caused higher tryptophan concentrations in comparison to control (Table 6).

Chloramine T-induced glycation resulted in significantly higher levels of amyloid-*β* structure, fructosamine, and AGE. The analysis showed significantly lower concentrations of fructosamine and AGE when all investigated inhibitors were added. Moreover, a statistically decreased amount of amyloid-*β* structure in samples with valsartan (95% vs. control), NAC (87% vs. control), and captopril (95% vs. control) was noticed. However, the BSA + chloramine T + metformin sample presented a significantly higher amyloid-*β* structure than the control (Table 6).

Significantly decreased antioxidant activity markers were observed when BSA was incubated with chloramine T. The addition of valsartan (106%, 119%, and 133% vs. control, respectively) and other investigated agents, except metformin, caused a significant increase of TAC, DPPH, and FRAP value (Table 6).

## 4. Discussion

Cardiovascular diseases (CVD) are the most common cause of death worldwide. Many factors are involved in the pathogenesis of such diseases, but the role of oxidative stress seems to be crucial [55–58]. Oxidative stress, which is defined as excessive production of reactive oxygen species (ROS) and disproportion of oxidants over antioxidants, causes myocardial remodeling by activating hypertrophy-signaling kinases and stimulating cardiac fibroblasts to proliferate. ROS also affect myocardial calcium handling and lead to cellular dysfunction by inducing changes in intracellular pathways [59, 60]. Moreover, in dysfunctional myocardium, enhanced ROS production is observed, and the antioxidant mechanism's exhaustion leads to disease progression [61, 62]. Oxidative stress likewise participates in the pathogenesis of hypertension. ROS decrease the availability of nitric oxide (NO), causing vasoconstriction and lead to modification of low-density lipoprotein (LDL), increasing its uptake by macrophages. As a result, the so-called foam cells are formed. Those cells take a very important part in atherosclerosis's pathogenesis, which is a well-known factor of hypertension and other diseases, such as coronary artery disease [16]. Oxidative stress is also a proven link between diabetes mellitus (DM) and CVD [63]. Furthermore, in patients diagnosed with DM, elevated levels of advanced glycation end products (AGE) are observed. This, along with oxidative stress, may explain the pathogenesis of cardiovascular complications of DM [64–67]. AGE are also a proven factor leading to heart failure and other CVD [11, 68–71].

Valsartan is a potent and specific angiotensin II receptor antagonist used to treat hypertension and chronic heart failure. Many clinical studies confirm the effectiveness of the drug, especially in combination with sacubitril [72, 73]. What is interesting, it is also proven that the therapy with valsartan significantly increases the long-term quality of life in patients with chronic heart failure [74], which is a result of biochemical, echocardiographic, and clinical improvements [75]. Simultaneously, the drug remains safe in patients with many comorbidities, especially in chronic kidney disease [76]. In patients with hypertension, the use of valsartan is also associated with the reduction of the risk of organ complications, including left ventricular hypertrophy [77, 78]. Due to the valsartan's pleiotropic properties, it is believed that the mechanism of drug action is not fully understood.

The research about the antioxidant properties of valsartan is especially limited. Nevertheless, the majority of accessible papers uphold the hypothesis of the drug's positive impact on the redox homeostasis *in vivo* [29, 79–90] (Table 7). Experiments conducted on animals show a statistically significant decrease in oxidative stress parameters and inflammatory and cellular damage markers. Valsartan also causes an increase in antioxidant enzymes' activities and decrease concentrations of adhesive and chemotactic factors. What is important, results of clinical studies remain consistent with the animal model results (Table 7). However, there are no data on the antiglycation activity of valsartan. Only Komiya et al. revealed a statistically significant decrease in blood AGE concentration in diabetic and hypertensive patients treated with valsartan [86]. This may suggest a potential antiglycation role, but there is no more available evidence to support this statement.

Despite all the facts that stand for valsartan as the antioxidant potential, some limitations are worth mentioning regarding this research. Available clinical studies were conducted in relatively small groups of patients, making it more challenging to perform a reliable statistical prediction. In some studies, patients continued the treatment with previously prescribed drugs before administering valsartan. It is unclear if these drugs had no known impact on cellular redox balance and interfere with the trial's results. There is also even more limited information regarding the influence of valsartan on protein glycooxidation. Thus, it is necessary to evaluate the effect of valsartan on protein oxidative damage by measuring the oxidation rate of thiol and carbonyl groups, aromatic amino acid residues, or assessing the extent of early and late protein glycation products.

Our study is the first to assess valsartan's antiglycation properties with respect to various glycating and oxidizing agents. Using an *in vitro* model, we have shown that valsartan strengthens the antioxidant barrier and inhibits oxidation and albumin glycation comparable to recognized ROS scavengers (Trolox, N-acetylcysteine, lipoic acid, and captopril) and protein glycation inhibitors (aminoguanidine and metformin). Considering the key role of oxidative stress in CVD pathology, valsartan's pleiotropic activity may result from its antioxidant and antiglycation properties.

Albumin, the main plasma protein, has a crucial role in the human organism. It is responsible for the transport of various substances such as hormones or drugs and maintaining blood pH or colloid-oncotic pressure. Albumin can also bind transition metal ions which explains its antioxidative properties [94–96]. Due to its high plasma concentration, long half-life, and high content of arginine, cysteine, and lysine, albumin can be easily glycated and oxidized *in vivo* [97, 98]. Albumin glycation involves nonenzymatic addition of reducing sugar to its amino groups. This process is subdivided into several phases. During the early ones are formed, the first Maillard reaction produces the Schiff base and the Amadori products. At the final stage, the advanced glycation end products (AGEs) are produced—including carboxymethyl lysine (CML), furyl-furanyl-imidazole (FFI), pentosidine, and pyralin [68, 70, 82, 98]. The glycation process occurs simultaneously with oxidation, by which they are collectively referred to as glycooxidation. The final products of protein oxidation are advanced oxidation protein products (AOPP). AOPPs originate from the accumulation of oxidized residue of arginine, cysteine, dityrosine, and tryptophan [69, 99, 100].

Our research demonstrated that valsartan ameliorates protein oxidation (↑total thiols, ↓PC, and ↓AOPP), reduces albumin glycooxidative damage (↑tryptophan, ↓kynurenine, ↓N-formylkynurenine, and ↓dityrosine), prevents glycation (↓amyloid-beta structure, ↓fructosamine, ↓and AGE), and enhances the antioxidant activity of albumin (↑TAC, ↑DPPH, and ↑FRAP). These results are similar in every investigated glycating or oxidizing agent combined with the drug. However, some differences in the drug's action are distinguishable between sugars and aldehydes. Generally, the antiglycooxidant properties of valsartan are more marked in samples containing GO or MGO and valsartan, where the parameters decrease or increase even more in comparison to the sugars samples (e.g., AGE content is lower in BSA + GO/MGO + valsartan than in BSA + Glu/Fru/Rib + valsartan). Nevertheless, it is worth mentioning that the oxidation and glycation substances used in the research were at greater concentrations than physiological levels that might impact the experiment's outcome [31, 32]. However, our model is kinetically validated and allows us to evaluate unknown substances' antiglycooxidative properties rapidly [31, 34]. There were no prominent differences between used sugars, as given results in their samples were comparable. Interestingly, valsartan exhibited antioxidant and antiglycooxidant properties in chloramine T presence, but glycation inhibition was not so potent as in the case of sugars or aldehydes.

Although our study does not explain it, valsartan's antiglycooxidant properties may be due to the molecule's chemical structure. It can be speculated that the -NH_2_ group in valsartan competes with the amino residues of proteins for the attachment of reactive carbonyl groups. Thus, it protects the lysine and arginine residues of proteins from their glycooxidative modifications. Therefore, future investigations are necessary to validate conclusions that can be drawn from this study. The next phase of research is the *in vivo* analysis of antiglycooxidant activity and optimal dosage of valsartan in animal and human models. However, the pleiotropic properties of valsartan may also be due to its other *in vivo* activities. Angiotensin II is a major effector peptide of the renin-angiotensin-aldosterone system, and it is generated from angiotensin I by the angiotensin-converting enzyme. Moreover, angiotensin II by acting on the AT_1_ receptor stimulates NADPH oxidase activation (Nox) and increases expression of Nox subunits leading to ROS overproduction [101]. Importantly, blockade of the renin-angiotensin system with blockers of AT_1_ receptors (ARBs) (such as valsartan) can reduce oxidative stress due to inhibition of the processes mentioned above. It was also shown that valsartan causes increased SOD expression, which transforms superoxide radicals into hydrogen peroxide and oxygen in a disproportionate reaction [102]. It is also speculated that ARBs can decrease protein oxidation via free radical scavenging and transition metal chelation [103].

Inhibition of protein glycation by valsartan may be of particular importance in patients with diabetic cardiomyopathy. AGE generated under hyperglycemic conditions increase ROS overproduction, which impairs the activity of ion pumps and mitochondria, disrupts the transport of calcium ions between cellular compartments, and initiates apoptosis. Extracellular matrix collagen also undergoes glycation, increasing cardiac stiffness and decreasing diastolic capacity and nerve impulse conduction velocity [61, 62]. It can be speculated that valsartan may inhibit the synthesis of AGE by directly neutralizing reactive dicarbonyl compounds by combining drug amino group with the *α*-dicarbonyl group of methylglyoxal. Nevertheless, these hypotheses require confirmation in further studies.

Overall, conducted research showed that valsartan can reduce oxidation and glycation damage and improve the albumin's antioxidant properties. The valsartan's activity was recorded against various oxidizing and glycating agents. What is more, the drug's action is comparable to many renowned antioxidants or is even more pronounced. The pleiotropic properties of valsartan may be due to its antiglycooxidant activity. Inhibition of protein glycation/oxidation in patients with CVD and DM may be crucial, given the significant contribution of oxidative and carbonyl stress to the development of cardiometabolic complications. Further studies considering this subject may be revolutionary for the treatment of cardiovascular diseases. Confirmation of the antiglycating effect of valsartan in human studies could result in a revision of clinical guidelines for the use of hypotensive drugs. Valsartan could be a first-line medicine in patients with heart disease and diabetes. Modifications to the chemical structure of valsartan should also be considered. The introduction of -NH_2_ groups (as in polyamide compounds) could increase the antiglycation activity's potency.

## Figures and Tables

**Table 1:** The effects of valsartan, Trolox, NAC, ALA, captopril, aminoguanidine, or metformin addition to BSA + glucose solution on protein oxidation, glycooxidation, and antioxidant activity.

	BSA	BSA + Glu	BSA + Glu + valsartan	BSA + Glu + Trolox	BSA + Glu + NAC	BSA + Glu + ALA	BSA + Glu + captopril	BSA + Glu + aminoguanidine	BSA + Glu + metformin
Protein oxidation									
Total thiols	108 ± 5.50	100	103 ± 2.20	109 ± 4.30∗	119 ± 0.95∗∗∗∗	105 ± 1.10∗∗	110 ± 7.90	116 ± 4.50∗∗	102 ± 1.20
PC	68 ± 3.30∗∗∗∗	100	84 ± 3.00∗∗∗	101 ± 2.70	76 ± 2.10∗∗∗∗	69 ± 1.80∗∗∗∗	78 ± 3.70∗∗∗	77 ± 5.50∗∗	98 ± 1.40
AOPP	66 ± 0.92∗∗∗∗	100	79 ± 1.50∗∗∗∗	66 ± 2.80∗∗∗∗	76 ± 3.20∗∗∗	76 ± 1.20∗∗∗∗	77 ± 3.00∗∗∗	68 ± 1.60∗∗∗∗	97 ± 1.70
Protein glycooxidation									
Tryptophan	127 ± 3.10∗∗∗	100	114 ± 2.20∗∗∗	132 ± 6.30∗∗∗	132 ± 6.30∗∗∗∗	137 ± 1.60∗∗∗∗	146 ± 0.43∗∗∗∗	125 ± 3.40∗∗∗	121 ± 2.00∗∗∗∗
Kynurenine	67 ± 0.35∗∗∗∗	100	70 ± 1.80∗∗∗∗	64 ± 0.38∗∗∗∗	71 ± 1.70∗∗∗∗	75 ± 1.60∗∗∗∗	72 ± 2.00∗∗∗∗	56 ± 1.30∗∗∗∗	79 ± 4.30∗∗
N-Formylkynurenine	66 ± 1.30∗∗∗∗	100	71 ± 3.00∗∗∗∗	65 ± 2.20∗∗∗∗	82 ± 0.38∗∗∗∗	80 ± 0.93∗∗∗∗	72 ± 0.44∗∗∗∗	44 ± 2.40∗∗∗∗	65 ± 2.60∗∗∗∗
Dityrosine	52 ± 5.90∗∗∗	100	55 ± 2.00∗∗∗∗	70 ± 1.60∗∗∗∗	51 ± 4.80∗∗∗∗	55 ± 5.20∗∗∗	68 ± 2.70∗∗∗∗	50 ± 5.40∗∗∗∗	90 ± 3.30∗∗
Albumin glycation									
Amyloid-*β* structure	79 ± 1.50∗∗∗∗	100	89 ± 1.30∗∗∗	81 ± 0.91∗∗∗∗	79 ± 2.00∗∗∗∗	82 ± 0.87∗∗∗∗	81 ± 1.50∗∗∗∗	68 ± 0.45∗∗∗∗	100 ± 1.50
Fructosamine	56 ± 2.40∗∗∗∗	100	56 ± 1.20∗∗∗∗	71 ± 1.70∗∗∗∗	61 ± 5.10∗∗∗	63 ± 4.40∗∗∗	67 ± 2.30∗∗∗∗	67 ± 2.00∗∗∗∗	85 ± 2.10∗∗∗
AGE	74 ± 0.82∗∗∗∗	100	73 ± 1.90∗∗∗∗	72 ± 0.83∗∗∗∗	72 ± 1.2∗∗∗	74 ± 1.6∗∗∗∗	82 ± 1.60∗∗∗	70 ± 0.56∗∗∗∗	89 ± 3.20
Albumin antioxidant activity									
TAC	122 ± 2.70∗∗∗	100	107 ± 2.90∗	102 ± 3.30	114 ± 2.40∗∗∗	105 ± 3.70	99 ± 6.30	110 ± 4.10∗	103 ± 3.70
DPPH	118 ± 2.10∗∗∗	100	109 ± 1.80∗∗	109 ± 0.14∗∗∗∗	128 ± 1.40∗∗∗∗	115 ± 4.30∗∗	111 ± 1.70∗∗∗	111 ± 2.20∗∗	104 ± 3.70
FRAP	123 ± 3.20∗∗∗	100	105 ± 2.00∗	113 ± 1.80∗∗∗	122 ± 2.30∗∗∗∗	111 ± 2.70∗∗	114 ± 0.80∗∗∗∗	113 ± 0.37∗∗∗∗	102 ± 1.10

Abbreviations: AGE: advanced glycation end products; ALA: alpha-lipoic acid; AOPP: advanced oxidation protein products; BSA: bovine serum albumin; DPPH: 2,2-di-phenyl-1-picrylhydrazyl radical scavenging capacity; FRAP: ferric reducing antioxidant power; Glu: glucose; NAC: N-acetylcysteine; PC: protein carbonyls; TAC: total antioxidant capacity; ∗*p* < 0.05 vs.control; ∗∗*p* < 0.01 vs. control; ∗∗∗*p* < 0.001 vs. control; ∗∗∗∗*p* < 0.0001 vs. control.

**Table 2:** The effects of valsartan, Trolox, NAC, ALA, captopril, aminoguanidine, or metformin addition to BSA + fructose solution on protein oxidation, glycooxidation, and antioxidant activity.

	BSA	BSA + Fru	BSA + Fru + valsartan	BSA + Fru + Trolox	BSA + Fru + NAC	BSA + Fru + ALA	BSA + Fru + captopril	BSA + Fru + aminoguanidine	BSA + Fru + metformin
Protein oxidation									
Total thiols	114 ± 0.63∗∗∗∗	100	105 ± 1.50∗∗	101 ± 4.80	114 ± 1.70∗∗∗	107 ± 1.40∗∗	101 ± 1.30	105 ± 1.10∗∗	102 ± 2.10
PC	62 ± 3.70∗∗∗∗	100	81 ± 1.40∗∗∗∗	70 ± 5.10∗∗∗	75 ± 4.70∗∗∗	65 ± 7.50∗∗	60 ± 2.70∗∗∗∗	56 ± 1.70∗∗∗∗	90 ± 2.60∗∗
AOPP	22 ± 0.89∗∗∗∗	100	58 ± 2.10∗∗∗∗	49 ± 0.62∗∗∗∗	39 ± 3.30∗∗∗∗	39 ± 1.20∗∗∗∗	45 ± 5.20∗∗∗∗	36 ± 3.20∗∗∗∗	95 ± 4.20
Protein glycooxidation									
Tryptophan	238 ± 2.40∗∗∗∗	100	100 ± 2.50	116 ± 12.00	103 ± 8.80	119 ± 12.00∗	158 ± 13.00∗	161 ± 3.60∗∗∗∗	107 ± 9.20
Kynurenine	57 ± 1.80∗∗∗∗	100	67 ± 1.80∗∗∗∗	68 ± 1.10∗∗∗∗	71 ± 3.10∗∗∗∗	53 ± 9.10∗∗∗	62 ± 2.90∗∗∗∗	66 ± 0.26∗∗∗∗	136 ± 3.40∗∗∗∗
N-Formylkynurenine	21 ± 1.80∗∗∗∗	100	57 ± 1.20∗∗∗∗	53 ± 0.21∗∗∗∗	59 ± 1.10∗∗∗∗	45 ± 0.01∗∗∗∗	71 ± 0.34∗∗∗∗	28 ± 1.80∗∗∗∗	187 ± 3.00∗∗∗∗
Dityrosine	48 ± 1.50∗∗∗∗	100	65 ± 1.90∗∗∗∗	67 ± 0.23∗∗∗∗	63 ± 1.20∗∗∗∗	71 ± 1.20∗∗∗∗	57 ± 0.78∗∗∗∗	54 ± 0.98∗∗∗∗	87 ± 1.00∗∗∗∗
Albumin glycation									
Amyloid-*β* structure	61 ± 0.13∗∗∗∗	100	88 ± 2.60∗∗	87 ± 0.34∗∗∗∗	86 ± 0.27∗∗∗∗	81 ± 0.47∗∗∗∗	81 ± 0.41∗∗∗∗	80 ± 1.90∗∗∗∗	108 ± 1.20∗∗∗
Fructosamine	51 ± 1.80∗∗∗∗	100	53 ± 3.50∗∗∗∗	55 ± 4.00∗∗∗∗	57 ± 4.00∗∗∗∗	55 ± 7.60∗∗∗	66 ± 3.90∗∗∗	62 ± 1.20∗∗∗∗	134 ± 2.80∗∗∗∗
AGE	59 ± 1.10∗∗∗∗	100	84 ± 1.60∗∗∗∗	81 ± 0.69∗∗∗∗	81 ± 0.29∗∗∗∗	85 ± 2.20∗∗∗	82 ± 0.58∗∗∗∗	77 ± 2.50∗∗∗∗	114 ± 2.10∗∗∗
Albumin antioxidant activity									
TAC	115 ± 1.10∗∗∗∗	100	97 ± 2.40	86 ± 9.00	114 ± 3.20∗∗	89 ± 7.10	94 ± 4.60	93 ± 5.60	94 ± 4.50
DPPH	145 ± 4.60∗∗∗∗	100	126 ± 2.40∗∗∗∗	130 ± 3.00∗∗∗∗	146 ± 3.10∗∗∗∗	129 ± 8.40∗∗	133 ± 3.00∗∗∗∗	124 ± 3.70∗∗∗	109 ± 3.20∗∗
FRAP	127 ± 2.30∗∗∗∗	100	104 ± 1.80∗	95 ± 3.30∗	129 ± 3.00∗∗∗∗	108 ± 1.80∗∗	108 ± 2.30∗∗	104 ± 2.40	98 ± 3.00

Abbreviations: AGE: advanced glycation end products; ALA: alpha-lipoic acid; AOPP: advanced oxidation protein products; BSA: bovine serum albumin; DPPH: 2,2-di-phenyl-1-picrylhydrazyl radical scavenging capacity; FRAP: ferric reducing antioxidant power; Fru: fructose; NAC: N-acetylcysteine; PC: protein carbonyls; TAC: total antioxidant capacity; ∗*p* < 0.05 vs. control; ∗∗*p* < 0.01 vs. control; ∗∗∗*p* < 0.001 vs. control; ∗∗∗∗*p* < 0.0001 vs. control.

**Table 3:** The effects of valsartan, Trolox, NAC, ALA, captopril, aminoguanidine, or metformin addition to BSA + ribose solution on protein oxidation, glycooxidation, and antioxidant activity.

	BSA	BSA + Rib	BSA + Rib + valsartan	BSA + Rib + Trolox	BSA + Rib + NAC	BSA + Rib + ALA	BSA + Rib + captopril	BSA + Rib + aminoguanidine	BSA + Rib + metformin
Protein oxidation									
Total thiols	128 ± 1.8∗∗∗∗	100	105 ± 2.1∗	106 ± 2∗∗	107 ± 0.82∗∗∗	104 ± 0.88∗∗	107 ± 2∗∗	109 ± 2.3∗∗	85 ± 2.9∗∗∗
PC	65 ± 1.2∗∗∗∗	100	68 ± 2.1∗∗∗∗	82 ± 1.7∗∗∗∗	69 ± 4.1∗∗∗	86 ± 6.3∗	69 ± 4.9∗∗∗	46 ± 1.7∗∗∗∗	90 ± 2.2∗∗
AOPP	58 ± 1.5∗∗∗∗	100	76 ± 2∗∗∗∗	81 ± 2∗∗∗∗	82 ± 1.5∗∗∗∗	75 ± 1.3∗∗∗∗	81 ± 2∗∗∗∗	63 ± 2.9∗∗∗∗	87 ± 2.7∗∗∗
Protein glycooxidation									
Tryptophan	132 ± 1.9∗∗∗∗	100	114 ± 1.4∗∗∗∗	103 ± 1.2∗	105 ± 0.1∗∗∗∗	113 ± 0.86∗∗∗∗	106 ± 1∗∗∗	118 ± 1.9∗∗∗∗	102 ± 1
Kynurenine	46 ± 1.2∗∗∗∗	100	83 ± 2.5∗∗∗	87 ± 0.36∗∗∗∗	77 ± 0.25∗∗∗∗	85 ± 0.98∗∗∗∗	86 ± 0.44∗∗∗∗	75 ± 1.2∗∗∗∗	96 ± 2.5
N-Formylkynurenine	44 ± 2.1∗∗∗∗	100	76 ± 2.8∗∗∗	81 ± 3.5∗∗∗	64 ± 1.8∗∗∗∗	76 ± 1.7∗∗∗∗	65 ± 1.2∗∗∗∗	63 ± 1.6∗∗∗∗	68 ± 1.5∗∗∗∗
Dityrosine	45 ± 4.2∗∗∗∗	100	68 ± 1.9∗∗∗∗	72 ± 1.6∗∗∗∗	67 ± 1.6∗∗∗∗	73 ± 1.9∗∗∗∗	77 ± 0.04∗∗∗∗	65 ± 1.2∗∗∗∗	101 ± 4.8
Albumin glycation									
Amyloid-*β* structure	59 ± 2.2∗∗∗∗	100	96 ± 1.7∗	96 ± 1.7∗	93 ± 1.4∗∗	95 ± 1.5∗∗	93 ± 1.2∗∗∗	94 ± 1.2∗∗∗	101 ± 4.2
Fructosamine	28 ± 2.4∗∗∗∗	100	58 ± 1.8∗∗∗∗	70 ± 8.6∗∗	75 ± 1.5∗∗∗∗	78 ± 2.1∗∗∗∗	60 ± 7∗∗∗	59 ± 3.2∗∗∗∗	94 ± 1.5∗∗
AGE	44 ± 1.8∗∗∗∗	100	66 ± 1.1∗∗∗∗	58 ± 1.1∗∗∗∗	61 ± 1.7∗∗∗∗	67 ± 1.7∗∗∗∗	60 ± 1.1∗∗∗∗	62 ± 1.2∗∗∗∗	77 ± 0.68∗∗∗∗
Albumin antioxidant activity									
TAC	132 ± 0.3∗∗∗∗	100	111 ± 3.1∗∗	118 ± 5.4∗∗	129 ± 4.4∗∗∗	104 ± 5.2	125 ± 3.6∗∗∗	111 ± 3.6∗∗	104 ± 3.4
DPPH	150 ± 3.8∗∗∗∗	100	119 ± 5.6∗∗	106 ± 0.89∗∗∗	150 ± 6.1∗∗∗	116 ± 8.1∗	108 ± 1.7∗∗	118 ± 0.93∗∗∗∗	95 ± 5.7
FRAP	135 ± 2.2∗∗∗∗	100	106 ± 0.54∗∗∗∗	108 ± 2.4∗∗	117 ± 1.1∗∗∗∗	110 ± 5∗	110 ± 1.6∗∗∗	105 ± 1.3∗∗	78 ± 7∗∗

Abbreviations: AGE: advanced glycation end products; ALA: alpha-lipoic acid; AOPP: advanced oxidation protein products; BSA: bovine serum albumin; DPPH: 2,2-di-phenyl-1-picrylhydrazyl radical scavenging capacity; FRAP: ferric reducing antioxidant power; NAC: N-acetylcysteine; PC: protein carbonyls; Rib: ribose; TAC: total antioxidant capacity; ∗*p* < 0.05 vs. control; ∗∗*p* < 0.01 vs. control; ∗∗∗*p* < 0.001 vs. control; ∗∗∗∗*p* < 0.0001 vs. control.

**Table 4:** The effects of valsartan, Trolox, NAC, ALA, captopril, aminoguanidine, or metformin addition to BSA + glyoxal solution on protein oxidation, glycooxidation, and antioxidant activity.

	BSA	BSA + GO	BSA + GO + valsartan	BSA + GO + Trolox	BSA + GO + NAC	BSA + GO + ALA	BSA + GO + captopril	BSA + GO + aminoguanidine	BSA + GO + metformin
Protein oxidation									
Total thiols	123 ± 2.80∗∗∗	100	103 ± 1.40∗	108 ± 3.00∗	118 ± 3.40∗∗∗	98 ± 2.70	114 ± 3.70∗∗	114 ± 3.70∗∗	102 ± 1.30∗
PC	64 ± 1.10∗∗∗∗	100	68 ± 2.20∗∗∗∗	89 ± 2.80∗∗	56 ± 4.10∗∗∗∗	85 ± 5.40∗∗	67 ± 1.40∗∗∗∗	67 ± 1.40∗∗∗∗	86 ± 2.80∗∗∗
AOPP	32 ± 2.00∗∗∗∗	100	40 ± 1.60∗∗∗∗	52 ± 3.50∗∗∗∗	32 ± 4.10∗∗∗∗	41 ± 4.00∗∗∗∗	50 ± 1.60∗∗∗∗	33 ± 2.40∗∗∗∗	64 ± 1.70∗∗∗∗
Protein glycooxidation									
Tryptophan	126 ± 1.30∗∗∗∗	100	113 ± 1.50∗∗∗	126 ± 3.40∗∗∗	113 ± 1.90∗∗∗	111 ± 2.80∗∗	112 ± 5.00∗	107 ± 1.60∗∗	103 ± 1.70∗
Kynurenine	65 ± 3.60∗∗∗∗	100	83 ± 2.20∗∗∗	82 ± 1.90∗∗∗∗	78 ± 2.00∗∗∗∗	96 ± 2.90	75 ± 4.30∗∗∗	62 ± 0.40∗∗∗∗	98 ± 0.48∗∗
N-Formylkynurenine	16 ± 2.30∗∗∗∗	100	42 ± 2.80∗∗∗∗	47 ± 3.60∗∗∗∗	38 ± 1.60∗∗∗∗	59 ± 2.20∗∗∗∗	37 ± 4.50∗∗∗∗	36 ± 3.00∗∗∗∗	65 ± 3.90∗∗∗
Dityrosine	25 ± 1.20∗∗∗∗	100	72 ± 1.50∗∗∗∗	55 ± 2.30∗∗∗∗	46 ± 1.40∗∗∗∗	68 ± 1.00∗∗∗∗	42 ± 0.78∗∗∗∗	26 ± 2.20∗∗∗∗	68 ± 1.70∗∗∗∗
Albumin glycation									
Amyloid-*β* structure	69 ± 0.72∗∗∗∗	100	71 ± 1.80∗∗∗∗	68 ± 0.79∗∗∗∗	66 ± 1.50∗∗∗∗	75 ± 1.80∗∗∗∗	62 ± 1.30∗∗∗∗	63 ± 1.90∗∗∗∗	83 ± 1.80∗∗∗∗
Fructosamine	66 ± 2.60∗∗∗∗	100	87 ± 2.90∗∗	92 ± 4.30∗	75 ± 3.40∗∗∗	86 ± 2.70∗∗∗	76 ± 2.60∗∗∗∗	85 ± 3.20∗∗	95 ± 3.40
AGE	23 ± 2.00∗∗∗∗	100	52 ± 1.60∗∗∗∗	68 ± 5.00∗∗∗	40 ± 1.80∗∗∗∗	61 ± 1.80∗∗∗∗	37 ± 4.80∗∗∗∗	35 ± 3.40∗∗∗∗	47 ± 3.20∗∗∗∗
Albumin antioxidant activity									
TAC	154 ± 7.20∗∗∗	100	116 ± 3.70∗∗	108 ± 0.97∗∗∗	156 ± 3.00∗∗∗∗	119 ± 2.30∗∗∗	108 ± 3.10∗	133 ± 4.20∗∗∗	111 ± 3.00∗∗
DPPH	152 ± 2.00∗∗∗∗	100	115 ± 2.10∗∗∗	128 ± 8.40∗∗	146 ± 3.50∗∗∗∗	130 ± 2.90∗∗∗∗	110 ± 6.80	133 ± 2.70∗∗∗∗	105 ± 1.40∗∗
FRAP	159 ± 5.50∗∗∗∗	100	118 ± 2.30∗∗∗	104 ± 5.30	117 ± 1.10∗∗∗∗	107 ± 1.00∗∗∗	88 ± 2.30∗∗∗	124 ± 2.40∗∗∗∗	78 ± 7.00∗∗

Abbreviations: AGE: advanced glycation end products; ALA: alpha-lipoic acid; AOPP: advanced oxidation protein products; BSA: bovine serum albumin; DPPH: 2,2-di-phenyl-1-picrylhydrazyl radical scavenging capacity; FRAP: ferric reducing antioxidant power; GO: glyoxal; NAC: N-acetylcysteine; PC: protein carbonyls; TAC: total antioxidant capacity; ∗*p* < 0.05 vs. control; ∗∗*p* < 0.01 vs. control; ∗∗∗*p* < 0.001 vs. control; ∗∗∗∗*p* < 0.0001 vs. control.

**Table 5:** The effects of valsartan, Trolox, NAC, ALA, captopril, aminoguanidine, or metformin addition to BSA + methylglyoxal solution on protein oxidation, glycooxidation, and antioxidant activity.

	BSA	BSA + MGO	BSA + MGO + valsartan	BSA + MGO + Trolox	BSA + MGO + NAC	BSA + MGO + ALA	BSA + MGO + captopril	BSA + MGO + aminoguanidine	BSA + MGO + metformin
Protein oxidation									
Total thiols	118 ± 0.3∗∗∗∗	100	106 ± 0.95∗∗∗	107 ± 1.1∗∗∗	109 ± 1.5∗∗∗	100 ± 1.4	107 ± 1.7∗∗	105 ± 1.1∗∗	97 ± 2
PC	57 ± 2.9∗∗∗∗	100	66 ± 1.8∗∗∗∗	71 ± 3.5∗∗∗	63 ± 1.5∗∗∗∗	58 ± 3.2∗∗∗∗	51 ± 2.5∗∗∗∗	48 ± 4∗∗∗∗	79 ± 4.4∗∗
AOPP	25 ± 1.2∗∗∗∗	100	55 ± 1.4∗∗∗∗	62 ± 2.5∗∗∗∗	55 ± 3.4∗∗∗∗	57 ± 0.71∗∗∗∗	53 ± 1.7∗∗∗∗	57 ± 1.8∗∗∗∗	90 ± 6.4
Protein glycooxidation									
Tryptophan	128 ± 1.1∗∗∗∗	100	107 ± 1.4∗∗	102 ± 0.16∗∗∗∗	100 ± 0.25	110 ± 0.31∗∗∗∗	103 ± 0.39∗∗∗	111 ± 0.15∗∗∗∗	102 ± 0.9∗∗
Kynurenine	41 ± 2.2∗∗∗∗	100	87 ± 2∗∗∗	95 ± 0.14∗∗∗∗	95 ± 1.3∗∗	99 ± 1.2	95 ± 3	76 ± 1.3∗∗∗∗	102 ± 0.25∗∗∗
N-formylkynurenine	13 ± 1.7∗∗∗∗	100	74 ± 1.9∗∗∗∗	60 ± 5.7∗∗∗	62 ± 4.1∗∗∗∗	93 ± 5.2	70 ± 7.1∗∗	46 ± 5.3∗∗∗∗	165 ± 6.1∗∗∗∗
Dityrosine	25 ± 3.9∗∗∗∗	100	72 ± 1.2∗∗∗∗	68 ± 4.6∗∗∗	65 ± 2.8∗∗∗∗	78 ± 5.6∗∗	70 ± 3.6∗∗∗	63 ± 2.5∗∗∗∗	102 ± 5.6
Albumin glycation									
Amyloid-*β* structure	32 ± 2.3∗∗∗∗	100	66 ± 1.9∗∗∗∗	86 ± 0.86∗∗∗∗	62 ± 1.2∗∗∗∗	50 ± 3∗∗∗∗	67 ± 2.2∗∗∗∗	52 ± 4.9∗∗∗∗	65 ± 1.3∗∗∗∗
Fructosamine	29 ± 1.2∗∗∗∗	100	38 ± 2.9∗∗∗∗	52 ± 5.2∗∗∗∗	30 ± 0.45∗∗∗∗	60 ± 6.1∗∗∗	40 ± 0.59∗∗∗∗	31 ± 6∗∗∗∗	39 ± 4.7∗∗∗∗
AGE	34 ± 3.5∗∗∗∗	100	56 ± 1.6∗∗∗∗	42 ± 2.5∗∗∗∗	42 ± 2.9∗∗∗∗	55 ± 3.2∗∗∗∗	46 ± 4∗∗∗∗	49 ± 6.2∗∗∗∗	80 ± 5.7∗∗∗∗
Albumin antioxidant activity									
TAC	219 ± 1.4∗∗∗∗	100	144 ± 0.93∗∗∗∗	107 ± 3.3∗	137 ± 7∗∗∗	137 ± 8.7∗∗	148 ± 3.7∗∗∗∗	141 ± 2.7∗∗∗∗	129 ± 6.3∗∗
DPPH	143 ± 4.4∗∗∗∗	100	113 ± 2.8∗∗	110 ± 3.1∗∗	127 ± 2.9∗∗∗∗	116 ± 8.2∗	106 ± 1.5∗∗	121 ± 2.9∗∗∗	104 ± 4.3
FRAP	150 ± 2.2∗∗∗∗	100	132 ± 1.7∗∗∗∗	105 ± 2.3∗	127 ± 3∗∗∗∗	127 ± 3.6∗∗∗	127 ± 4.9∗∗∗	123 ± 5.8∗∗	99 ± 4.2

Abbreviations: AGE: advanced glycation end products; ALA: alpha-lipoic acid; AOPP: advanced oxidation protein products; BSA: bovine serum albumin; DPPH: 2,2-di-phenyl-1-picrylhydrazyl radical scavenging capacity; FRAP: ferric reducing antioxidant power; MGO: methylglyoxal; NAC: N-acetylcysteine; PC: protein carbonyls; TAC: total antioxidant capacity; ∗*p* < 0.05 vs. control; ∗∗*p* < 0.01 vs. control; ∗∗∗*p* < 0.001 vs. control; ∗∗∗∗*p* < 0.0001 vs. control.

**Table 6:** The effects of valsartan, Trolox, NAC, ALA, captopril, aminoguanidine, or metformin addition to BSA + chloramine T solution on protein oxidation, glycooxidation, and antioxidant activity.

	BSA	BSA + chloramine T	BSA + chloramine T + valsartan	BSA + chloramine T + Trolox	BSA + chloramine T + NAC	BSA + chloramine T + ALA	BSA + chloramine T + captopril	BSA + chloramine T + aminoguanidine	BSA + chloramine T + metformin
Protein oxidation									
Total thiols	126 ± 0.36∗∗∗∗	100	110 ± 2.00∗∗∗	104 ± 1.10∗∗	111 ± 1.40∗∗∗	110 ± 0.64∗∗∗∗	113 ± 1.40∗∗∗∗	110 ± 1.20∗∗∗	115 ± 1.80∗∗∗
PC	57 ± 1.80∗∗∗∗	100	71 ± 2.40∗∗∗	64 ± 2.50∗∗∗∗	57 ± 4.70∗∗∗∗	58 ± 2.90∗∗∗∗	51 ± 3.50∗∗∗∗	54 ± 3.40∗∗∗∗	56 ± 6.90∗∗∗
AOPP	31 ± 0.63∗∗∗∗	100	56 ± 2.10∗∗∗∗	58 ± 2.80∗∗∗∗	31 ± 0.77∗∗∗∗	31 ± 1.10∗∗∗∗	64 ± 1.70∗∗∗∗	56 ± 5.60∗∗∗	85 ± 2.90∗
Protein glycooxidation									
Tryptophan	130 ± 0.58∗∗∗∗	100	101 ± 1.20∗∗∗∗	102 ± 0.12∗∗∗∗	104 ± 0.66∗∗∗	107 ± 0.58∗∗∗∗	97 ± 0.66∗∗	101 ± 1.30	97 ± 1.20∗
Kynurenine	36 ± 1.20∗∗∗∗	100	89 ± 2.00∗∗∗∗	88 ± 1.30∗∗∗	50 ± 1.90∗∗∗∗	78 ± 2.70∗∗∗	53 ± 1.20∗∗∗∗	91 ± 1.90∗∗	81 ± 2.40∗∗∗
N-Formylkynurenine	12 ± 1.80∗∗∗∗	100	69 ± 1.40∗∗∗∗	62 ± 1.80∗∗∗∗	46 ± 2.00∗∗∗∗	51 ± 0.74∗∗∗∗	54 ± 1.40∗∗∗∗	93 ± 2.00∗∗	47 ± 1.90∗∗∗∗
Dityrosine	46 ± 0.28∗∗∗∗	100	77 ± 1.40∗∗∗∗	82 ± 1.30∗∗∗∗	42 ± 1.20∗∗∗∗	53 ± 1.20∗∗∗∗	64 ± 1.30∗∗∗∗	82 ± 1.30∗∗∗∗	84 ± 1.90∗∗∗
Albumin glycation									
Amyloid-*β* structure	81 ± 0.41∗∗∗∗	100	95 ± 0.83∗∗	84 ± 1.30	87 ± 2.50∗∗∗	98 ± 1.10	95 ± 0.67∗∗∗	99 ± 0.94	112 ± 1.80∗∗∗
Fructosamine	61 ± 3.40∗∗∗∗	100	86 ± 0.43∗∗∗∗	66 ± 3.90∗∗∗	57 ± 1.30∗∗∗∗	79 ± 1.60∗∗∗∗	69 ± 3.50∗∗∗	87 ± 1.30∗∗∗∗	66 ± 2.60∗∗∗∗
AGE	44 ± 1.00∗∗∗∗	100	74 ± 2.60∗∗∗∗	74 ± 0.85∗∗∗∗	76 ± 1.70∗∗∗∗	79 ± 1.90∗∗∗∗	62 ± 0.05∗∗∗∗	91 ± 2.80∗∗	86 ± 0.97∗∗∗∗
Albumin antioxidant activity									
TAC	124 ± 2.90∗∗∗	100	106 ± 3.40∗∗∗	111 ± 1.40∗∗∗	116 ± 1.00∗∗∗∗	125 ± 3.60∗∗∗	116 ± 0.85∗∗∗∗	105 ± 2.90∗	103 ± 2.70
DPPH	157 ± 4.60∗∗∗∗	100	119 ± 3.00∗∗∗	157 ± 4.00∗∗∗∗	158 ± 2.70∗∗∗∗	137 ± 2.10∗∗∗∗	146 ± 4.20∗∗∗∗	113 ± 3.00∗∗	108 ± 2.90
FRAP	150 ± 2.20∗∗∗∗	100	133 ± 3.20∗∗∗∗	134 ± 3.80∗∗∗	167 ± 4.00∗∗∗∗	127 ± 5.10∗∗∗	126 ± 7.10∗∗	122 ± 5.30∗∗	107 ± 1.20

Abbreviations: AGE: advanced glycation end products; ALA: alpha-lipoic acid; AOPP: advanced oxidation protein products; BSA: bovine serum albumin; DPPH: 2,2-di-phenyl-1-picrylhydrazyl radical scavenging capacity; FRAP: ferric reducing antioxidant power; NAC: N-acetylcysteine; PC: protein carbonyls; TAC: total antioxidant capacity; ∗*p* < 0.05 vs. control; ∗∗*p* < 0.01 vs. control; ∗∗∗*p* < 0.001 vs. control; ∗∗∗∗*p* < 0.0001 vs. control.

**Table 7:** The pleiotropic properties of valsartan in experimental and clinical studies.

Valsartan properties	Study design	Measured parameters	Results	References
Valsartan presenting antioxidant properties in myocardial ischemia and myocardial infarction model in rats	Seven male albino rats pretreated intraperitoneally (i.p.) with valsartan (10 mg/kg) before left anterior descending artery (LAD) ligation vs. rats which undergo the same intervention, but with no pretreatment or saline i.p. injection	Cardiac troponin T (cTnT) in heart blood plasma	↓cTnT concentration in valsartan-pretreated group vs. control (*p* < 0.001)	Hadi et al. 2015 [79]
		Malondialdehyde (MDA) and reduced glutathione (GSH) in heart blood serum	↓MDA concentration in valsartan-pretreated group vs. control (*p* < 0.001)	
		Tumor necrosis factor (TNF), interleukin 6 (IL-6), interleukin 10 (IL-10), caspase 3, and BAX protein in cardiac tissue	↓TNF, ↓IL-6, ↓IL-10 ↓caspase 3, ↓BAX concentrations in valsartan-pretreated group vs. control (*p* < 0.001)	
		Histopathological study of cardiac tissue	Histopathological injure improvement in valsartan-pretreated rats vs. control (14.3% of the group had no signs of injury; *p* < 0.001)	
	16 male Sprague-Dawley rats treated with p.o. valsartan (10 mg/kg/d, 2 weeks) after ligation of LAD vs. 16 not pretreated rats that undergone the same procedure	Plasma MDA, superoxide dismutase (SOD) activity, and TNF-*α*	↓MDA, ↑SOD after 4 h of reperfusion vs. ischemia-reperfusion control group (*p* < 0.05)	Wu et al. 2013 [80]
		Myocardial nicotinamide adenine dinucleotide phosphate (NADPH) oxidase and nuclear factor–kappa B (NF-*κ*B)	↓TNF-*α* in 60 min, 120 min, and 240 min after reperfusion vs. ischemia-reperfusion control (*p* < 0.05)	
			↓NADPH oxidase activity, ↓NF-*κ*B expression vs. ischemia-reperfusion control (*p* < 0.05)	
	2 groups of 6 male Wistar rats premedicated with valsartan 50 mg/kg or 100 mg/kg for 14 days and then treated with isoproterenol (ISO) to induce MI	Total antioxidant activity (TAC) and nitric oxide (NO) in serum	↓1.6x/2.03x TAC (VAL 50/100, *p* < 0.01/*p* < 0.001) vs. MI-control	Imran et al. 2019 [81]
		Catalase, SOD, glutathione peroxidase (GPX), glutathione reductase (GR), glutathione S-transferase (GST), thiobarbituric acid-reactive substances (TBARS) in cardiac tissue	↓1.58x NO (VAL 100, *p* < 0.01)	
			↑ CAT (VAL 100, *p* < 0.05)	
			↑ SOD (VAL 100, *p* < 0.01)	
			↑2.7x/3.45x GPX	
			(VAL 50/100, *p* < 0.05/*p* < 0.05)	
		Histopathological study of cardiac tissue	↑2.26x/2.42x GR (VAL 50/100, *p* < 0.05)	
			↑1.86x/2.11x GST (VAL 50/100, *p* < 0.05/*p* < 0.01)	
			1.86x/2.29x ↓TBARS (VAL 50/100, *p* < 0.05/*p* < 0.01)	
			A few inflammatory cells and vacuolization in VAL 50 myocardium	
			Muscle fibers with no significant changes except for larger nucleus in VAL 100	
Valsartan ameliorates cardiac hypertrophy resulting from cardiac pressure overload in rats	4 groups of Sprague-Dawley rats (sham (*n* = 16), vehicle (*n* = 26), valsartan (*n* = 29), sacubitril/valsartan (*n* = 28)) undergoing aortic banding to induce cardiac pressure overload	Left ventricular weight	No significant change of left ventricular weight in VAL group vs. vehicle (*p* = 0.04)	Nordén et al. 2021 [91]
		Mean arterial pressure (MAP)	↓16% MAP in VAL group vs. sham	
		Atrial and brain natriuretic peptide (ANP, BNP)	↑ANP, BNP in VAL group vs. sham (*p* < 0.05)	
		Histopathological study of cardiac tissue	↑Expression of collagen 1, 3, and metalloproteinase-2 of cardiac tissue in VAL group	
Reduction of oxidative damage of kidneys with valsartan in streptozotocin-induced diabetes in rats	8 male Wistar rats with streptozotocin-induced diabetes treated with p.o. valsartan (100 mg/kg/d, 1 month) vs. 8 diabetic, nontreated rats	MDA, SOD, GPX, renal TNF-*α*-mRNA, renal TGF-*β*-mRNA, renal IL-6, NAD/NADH ratio in renal tissue homogenate	↓TNF-*α*-mRNA, ↓IL-6, ↓urine IL-6, ↓TGF-*β*-mRNA, ↑NAD/NADH ratio vs. diabetic rats control group (*p* < 0.001)	Sanajou et al. 2019 [82]
		IL-6 in urine	↓Renal MDA, ↑renal GPX, ↑renal SOD vs. diabetic rats control group (*p* < 0.001)	
		Histopathological study of renal tissue	Significant reduction of collagen deposition in the tubulointerstitial area	
Antiatherogenic effect of valsartan in cholesterol-fed rabbits	Male rabbits fed with normal (*n* = 4), cholesterol (*n* = 6), or cholesterol + valsartan (*n* = 5) diet (1 mg/kg, subcutaneously)	Systolic and diastolic blood pressure (SBP, DBP)	No significant change of SBP or DBP in VAL group vs. cholesterol-fed	Li et al. 2004 [92]
		Total cholesterol and triglycerides (TC, TG)	No significant change of TC in VAL group vs. cholesterol-fed	
		Serum ACE activity	↓TG in VAL group vs. cholesterol-fed (*p* < 0.05)	
		Morphology and histopathological studies of aorta	↓Of atherosclerotic lesion area in VAL group, but not statistically significant	
Inflammation and vascular remodeling attenuation in valsartan-treated diabetic and hyperlipidemic swine	24 diabetic and hyperlipidemic swine divided into three groups (early and late); placebo (*n* = 4), valsartan 320 mg daily (*n* = 4), valsartan + simvastatin 320 mg/40 mg (*n* = 4)	Progression of atherosclerotic lesions	Slower progression of atherosclerotic plaques vs. placebo	Chatzizisis et al. 2009 [93]
		Histopathological studies	↓area of inflammation in plaques in VAL group vs. placebo (*p* = 0.041)	
		Expression of extracellular matrix metalloproteinases (MMP-2, MMP-9)	↓MMP-9 ratio in plaques with expansive remodeling in VAL group (*p* = 0.1)	
		Expression of tissue inhibitors of metalloproteinases (TIMP-1, TIMP-2)	↓MMP/TIMP ratio in plaques with expansive remodeling in VAL group (*p* = 0.01)	
Antioxidant action of valsartan in high-glucose-cultured rat mesangial cells	HBZY-1 rat mesangial cells cultured with high glucose (30 mmol/lLconcentration and valsartan (10 *μ*mol/L) vs. cells cultured in high-glucose only vs. osmotic pressure control group (25 mmol/L of mannitol, 5 mmol/L of glucose)	SOD activity, nitric oxide (NO), and malondialdehyde (MDA) contents in supernatant	↑SOD activity and ↑NO, ↓MDA contents in valsartan + high-glucose group vs. high-glucose only group (after 24 hrs of incubation)	Liu et al. 2016 [83]
Oxidative stress parameters in type 2 diabetic and hypertensive patients treated with valsartan	33 patients with type 2 diabetes and hypertension treated with valsartan (80 mg/d) for 24 weeks (no other antihypertensive drugs 2 weeks before the screening date)	Nitrotyrosine in blood	↓Nitrotyrosine after 24 weeks of treatment (0.68 ± 0.59 nmol/L vs. 0.38 ± 0.39 nmol/L, *p* < 0.007)	Kim et al. 2017 [84]
	26 patients with hypertension (>140/90 mmHg) and mild diabetes (HbA_1c_ < 9%) treated with 40 or 80 mg/kg valsartan for 3 months, all previously taken drugs were continued	Blood inflammatory markers (hs-CRP, IL-6, IL-18, VCAM-1, L-selectin)	↓hs-CRP (0.231 vs. 0.134, *p* = 0.043)	Kuboki et al. 2007 [85]
			↓VCAM-1 (471.1 vs. 403.2, *p* = 0.012)	
			↑IL-6, IL-18, L-selectin (but statistically insignificant)	
		8-Isoprostane and 8-OHdG in urine	↓8-OHdG after treatment (12.12 vs. 8.07, *p* = 0.001)	
			↑8-Isoprostane after treatment (283.5 vs. 302.0, but statistically insignificant)	
	15 patients with type 2 diabetes treated with 40 mg/d valsartan (in 6 patients dose increased to 80 mg/d after 6 months) for 1 year	AGE in blood	↓AGE (2.71 ± 0.62 vs. 2.29 ± 0.28, *p* = 0.021)	Komiya et al. 2008 [86]
		Urine 8-isoprostane	↓8-Isoprostane (347 ± 215 vs. 205 ± 122, *p* = 0.025)	
Valsartan ameliorated oxidative stress in patients with hypertension	25 patients with hypertension (>140/90 mmHg) treated with 80 or 160 mg/d valsartan depending on basal arterial pressure	Urine 8-isoprostane and 8-hydroxy-2′-deoxyguanosine (8-OHG)	55% ↓urine 8-isoprostane and 30% ↓8-OHdG after 12 months of treatment (*p* < 0.05)	Hirooka et al. 2008 [29]
	17 patients with hypertension (>140/90 mmHg) treated with 40 or 80 mg/d valsartan if BP did not decrease below 140/90 mmHg	Serum levels of lipoperoxidation (LPO)	↓LPO after 6 months, when pretreatment LPO levels were ≥1.5 pg/mL (2.12 ± 0.23 vs. 1.15 ± 0.31, *p* < 0.05)	Miyajima et al. 2008 [87]
	8 patients with hypertension and hyperlipidemia treated with 80 mg/d valsartan for 2 months	Plasma TBARS	↓TBARS after the treatment (33.0 ± 2.8 vs. 26.6 ± 1.8, *p* < 0.05)	Hussein et al. 2002 [88]
		Lag time required for the initiation of LDL oxidation	↑Lag time after the treatment (57 min ± 3 vs. 70 min ± 6, *p* < 0.05)	
Valsartan improved an atherosclerotic lesion in mice by oxidative stress improvement	Male apoE-deficient mice on standard or high-cholesterol diet (HCD) treated with 0.5 mg/kg valsartan i.p. in osmotic minipump	Expression of NADPH oxidase subunit p47^phox^	58% ↓p47^phox^ in HCD + valsartan group vs. HCD-only (*p* < 0.05)	Suzuki et al. 2006 [89]
		The area of the atherosclerotic lesion in the aorta	2% of area in HCD + valsartan group vs. 5% in HCD group (*p* < 0.05)	
Effect of valsartan on redox balance in hemodialysed patients with end-stage renal disease	19 patients with end-stage renal disease treated with 320 mg/d valsartan for 6 weeks	Protein carbonyls (PC), 8-OHdG, 13-hydroxyoctadecadienoic acid (13-HODE), hs-CRP in plasma	No change in PC (0.17 ± 0.01 before and after, but statistically insignificant)	Aslam et al. 2006 [90]
		Glutathione disulfide: reduced glutathione ratio (GSSG:GSH) in whole blood	19.5% ↓ 8-OHdG (2.97 ± 0.22 vs. 2.38 ± 0.15, *p* < 0.05)	
			14.4% ↓13-HODE (428.3 ± 26.8 vs. 366.87 ± 15.8, but statistically insignificant)	
			↑hs-CRP (0.84 ± 0.36 vs. 1.33 ± 0.5, but statistically insignificant)	
			79% ↓GSSG: GSH (2.9 ± 3.1 vs. 0.6 ± 0.1)	

## Data Availability

The article contains complete data used to support the findings of this study.
